# A study by the lattice discrete element method for exploring the fractal nature of scale effects

**DOI:** 10.1038/s41598-022-20137-3

**Published:** 2022-10-06

**Authors:** Luis Eduardo Kosteski, Ignacio Iturrioz, Leandro Ferreira Friedrich, Giuseppe Lacidogna

**Affiliations:** 1 PPEng, Federal University of Pampa, Campus Alegrete, Alegrete, RS Brazil; 2 PROMEC, Federal Univerisity of Rio Grande do Sul, Porto Alegre, RS Brazil; 3 Department of Structural, Geotechnical and Building Engineering, Politecnico di Torino, Turin, Italy

**Keywords:** Engineering, Materials science

## Abstract

Nowadays, there are many applications in the field of Engineering related to quasi-brittle materials such as ceramics, natural stones, and concrete, among others. When damage is produced, two phenomena can take place: the damage produced governs the collapse process when working with this type of material, and its random nature rules the nonlinear behavior up to the collapse. The interaction among clouds of micro-cracks generates the localization process that implies transforming a continuum domain into a discontinue one. This process also governs the size effect, that is, the changes of the global parameters as the strength and characteristic strain and energies when the size of the structure changes. Some aspects of the scaling law based on the fractal concepts proposed by Prof Carpinteri are analyzed in this work. On the other hand, the Discrete Method is an interesting option to be used in the simulation collapse process of quasi-brittle materials. This method can allow failures with relative ease. Moreover, it can also help to relax the continuum hypothesis. In the present work, a version of the Discrete Element Method is used to simulate the mechanical behavior of different size specimens until collapse by analyzing the size effect represented by this method. This work presents two sets of examples. Its results allow the researchers to see the connection between the numerical results regarding the size effect and the theoretical law based on the fractal dimension of the parameter studied. Two main aspects appear as a result of the analysis presented here. Understand better some aspects of the size effect using the numerical tool and show that the Lattice Discrete Element Method has enough robustness to be applied in the nonlinear analysis of structures built by quasi-brittle materials.

## Introduction

Problems of practical interest in engineering in the most varied scales are inherently related to the knowledge of the damage evolution in quasi-brittle materials such as ceramics, concrete, and rocks, among others. These materials characterize the nonlinear mechanical behavior governed by clouds of micro-cracks that interact and grow in size and intensity.

However, the study of the damage evolution and the rupture in quasi-brittle materials is an area where consistent calculation methodologies do not yet exist^1^. A complex fracture process characterizes the collapse in quasi-brittle materials. This process must consider the scale effect when determining the mechanical parameters involved, the random nature of the mechanical properties, and the phenomenon of micro-cracks interaction. The micro-crack interacts in a complex way because it produces at the end of the damage process the nucleation of one or more macro-cracks that determine the rupture of the structure under study. Therefore, the fracture in these kinds of materials constitutes a cooperative and multiscale problem, and where the phenomenon becomes critical, other length scales start to play an important role^2^. For this reason, it is impossible to treat global behavior by means of a local law^3^.

Weibull^4^ first explains the scale effect based on the weakest link theory. He assumes that the material fails due to a critical flaw within a portion of the material under tensile load. At the same time, the probability of finding a critical flaw is lower in smaller samples and higher in larger samples. In seismology, for example, how large rock masses are damaged may be key to studying the formation and propagation of seismic waves, as emphasized in Refs.^5–7^. Ref.^8^ presents an excellent revision of the classical approach to dealing with the damage process since the problem is on a scale of meters to a scale of nanometers.

In Ref.^9^, the authors observe that self-similarity (the property of sets showing statistically similar morphologies at various scales of observation) is found on the fracture surface of many heterogeneous materials, such as rocks, concrete, and metals. They also emphasized that this aspect cannot be ignored or even replaced by a mean-field when analyzing materials with this type of disorder since many length scales interact during the material failure process. Therefore, Euclidian descriptions can no longer be used in modeling this type of problem and must be replaced by the fractal description that represents the fundamental character of the phenomenon from the physical and topological point of view. Thus, the material's behavior depends on the disorder and its relation with its size at the macro scale. Independent of the scale, the microstructural disorder for the same material becomes less important as the size increases. From a fractal point of view, this represents the change from a non-integer dimension to an integer dimension, that is, Euclidean space.

On the other hand, several numerical methodologies are available in the literature to study damage evolution in quasi-brittle materials. For example, the damage can be studied using classical methods of continuum mechanics, such as the work of Hilleborg et al*.*^10^. It is possible to modify this methodology to introduce the possibility of discrete cracks appearing in the material. Some examples of this are the Cohesive Zone Model (CZM) proposed by Xu and Needleman^11^. An extensive research production follows this approach, among others could be cited the work of Park and Paulino^12^. Other technics proposed originally by Belytschko and Black^13^ introduce the singularity of cracks as particular functions in interpolating displacements in the finite element method. This approach is called XFEM and, among other works, could cite Ref.^14^. Another widespread implementation in the context of FEM is the Phase Field Method (PFM) proposed by Francfort and Marigo^15^, with the numerical implementation present in Ref.^16^. Ambati et al*.*^17^ present a review of the PFM approach. The lack of these proposals is the difficulty of considering the random character of the material properties, as was pointed out by Krajcinovic^1^.

An alternative to the previously mentioned and criticized proposals is to use methods not based on continuum mechanics. These methods consist of formulations that present an arrangement of nodes with masses linked to them by interaction functions. These functions represent the equivalent stiffness of the structure. Among these methods, we can mention the Peridynamic proposal by Silling^18^. The method proposed by Silling has been employed in the last decade to simulate fracture and fragmentation, particularly in quasi-brittle materials, as described in Refs.^19–23^. Initially proposed by Riera^24^, the version of the Discrete Element Method used in the present work can be interpreted as a simplification of the Peridynamic approach or a version of the Lattice Model or Discrete Element approaches.

The Riera^24^ approach, referred to as the Lattice Discrete Element Method (LDEM), consists of a regular arrangement of discrete masses joined by bars with a regular cubic distribution. Explicit expressions allow determining the bar's equivalent stiffness to represent the simulated solid. The damage law of each bar naturally lets us describe the damage, the rupture, and the fragmentation, in this way, making the transition from continuous to discontinuous take place in a natural way. The time domain problem is solved by integrating a motion equation resulting from the spatial discretization performed. An explicit integration scheme is applied to the implementation used in this work. The random characteristics of the material are introduced in the model, considering some of the main properties as random fields. These properties can be the modulus of elasticity and/or the intrinsic fracture energy of the material. The parameter's probability distributions described by their mean, coefficient of variation, and spatial correlation define the random field. The method presented has been used successfully in the representation of concrete and reinforced concrete, as can be seen in Refs.^25,26^, and of particular characteristics of quasi-brittle materials such as their ability to emit acoustic emission signals during the fracture process^27–29^. Several works used this method to explore some of the ideas previously presented by Carpinteri, as in Refs.^30–34^, that involve topics directly or indirectly linked to the scale effect, fracture, acoustic emission, and fractality.

In the present work, the LDEM is used to analyze the scale effect in quasi-brittle material specimens. The results are analyzed using the theoretical frame proposed by Carpinteri et al.^35–37^ in its Fractal Size Effect theory. Different aspects of how this theory is verified and how to interpret some apparent incongruence between the LDEM and the Fractal Size Effect theory proposed by Carpinteri are shown. The discussions presented here have two goals: to understand better some aspects of the size effect using the numerical tool and to show that the LDEM has enough robustness to be applied in the nonlinear analysis of structures built by quasi-brittle materials.

## Scale-independent cohesive law

Carpinteri et al*.*^38^, using the concept of fractal dimensions introduced by Mandelbrot^39^, proposed a scale-independent cohesive law for quasi-brittle materials. To achieve this, he proposed three material parameters: the tensile strength *σ*_*u*_, the fracture energy *G*_*f*,_ and the critical strain *ε*_*c*_, which must be defined in a non-conventional form known as *fractal tensile strength σ*_u_^*^, *fractal fracture energy G*_f_^*^, and *fractal critical strain ε*_c_^*^, respectively. These parameters are true material constants, *i.e.,* they are scale-invariant material parameters and possess the anomalous physical dimensions $[F]{[L]}^{-(2-{d}_{\sigma })}$, ${[L]}^{({d}_{\varepsilon })}$ and $[FL]{[L]}^{-(2+{d}_{G})}$, respectively. Table 1 summarizes the scale effect of these properties described by power laws indicated in the bi-log domain^9, 35,40–45^.

**Table 1:** Summary of the power laws that represent the scale effect of tensile strength *σ*_*u*_, critical strain *ε*_*c*_ and fracture energy *G*_*f*_^9,35,40–45^.

${\sigma }_{u}\sim {\sigma }_{u}^{*}{b}^{-{d}_{\sigma }}$	${w}_{c}={\varepsilon }_{c}b={\varepsilon }_{c}^{*}{b}^{\left(1-{d}_{\varepsilon }\right)}$	${G}_{f}\sim {G}_{f}^{*}{b}^{{d}_{G}}$
		

In the expressions of Table 1, *b* is the characteristic size of the specimen and ${d}_{\sigma }$, ${d}_{\varepsilon }$ and ${d}_{G}$ are the fractal exponents of the tensile strength, critical strain and fracture energy, respectively. It is important to note that ${d}_{G}$ can be obtained as a function of ${d}_{\sigma }$ and ${d}_{\varepsilon }$. The following fundamental relation between the scale exponents results:

1 $${d}_{\sigma }+{d}_{\varepsilon }+{d}_{G}=1$$

Carpinteri^38^ proves that all the exponents are positive and smaller than 1 and also that the sum of ${d}_{\sigma }$ and ${d}_{G}$ is always smaller than 1, as shown by Carpinteri^36^ employing dimensional arguments.

**Figure 1:**
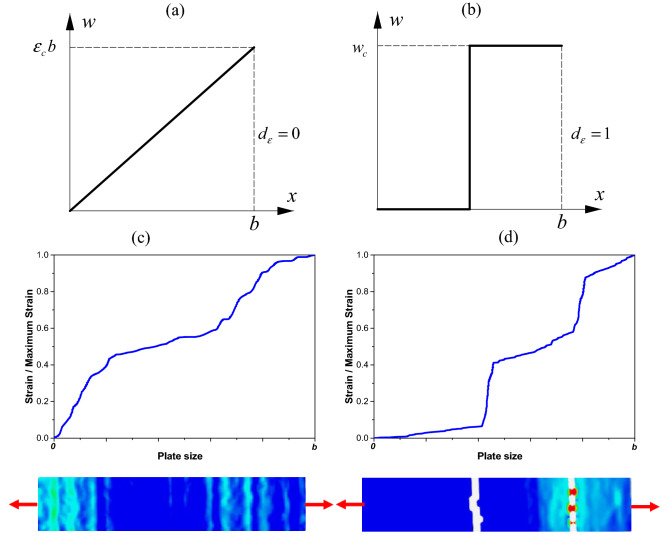
(**a**) Extremely diffused strain and (**b**) extremely localized deformation over the bar. (**c**) and (**d**) deformation field obtained by DIC of concrete specimens submitted to tensile load. In the strain color scale, blue is close to zero, and red is maximum up to the material break.

The fractional exponent ${d}_{\varepsilon }$ is intimately related to the degree of disorder in the mesoscopic damage process. It can vary between 1, corresponding to the homogeneous regime (large scale), and 0, corresponding to the fractal regime (small scale). When ${d}_{\varepsilon }=0$, the collapse is governed by the canonical critical strain *ε*_*c*_. In this case, for a simple stretched bar, the structure being studied has diffused damage and a ductile behavior (Fig. 1a). However, when ${d}_{\varepsilon }=1$, the collapse is governed by the critical displacement *w*_*c*_, which is size-dependent. In this case, the localization of the damage is placed onto a single cross-section, i.e., brittle behavior (Fig. 1b). To illustrate the same experimental tendency, Fig. 1c,d present two deformation fields of concrete specimens subjected to tensile traction obtained with Digital Image Correlation (DIC) of the tested samples. Figure 1c shows an image with a little dispersion near diffuse damage, and Fig. 1d shows the localization of deformation in two sections. The place that is not colored in Fig. 1d is featured that way because the deformations are big enough, and the DIC software cannot recognize this area anymore.

**Figure 2:**
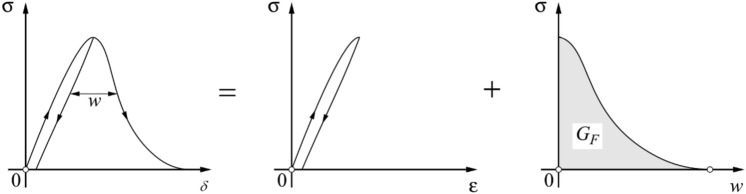
Experimental measure of fracture energy.

To find the fractal exponent ${d}_{G}$, it is important to mention first that there are more usual techniques for obtaining this value within the context of fractality. It can be obtained through direct observation of the fracture surface, with the patchwork method, box counting, or the spectral method, all described in greater detail in Refs.^45,46^. However, ${d}_{G}$ can also be obtained by the *G*_*f*_ values, found through the area of the stress-crack opening or displacement (*w*) curve, Fig. 2.

It is important to note that the area of the stress-displacement (*δ*) curve represents the total work done by the system. Thus, this area is the sum of all the energies involved in the rupture process of the sample. References^35,38,47^ define *w* as:

2 $$w={w}_{T}-{w}_{pp}$$

where *w*_*T*_ is the total displacement and ${w}_{pp}$ is the displacement due to elastic and inelastic deformations before attaining the peak stress in the test under examination.

It is possible to consider that at the beginning of the damage process, the energy dissipates from a volumetric region of the specimen. That is, from a dominium of 3D dimension. This situation happens in the case of the early stages of the damaging process over a body submitted to a homogeneous tensile stress field. Conversely, it is possible to consider that the energy dissipates from a region of 2D dimension when the main crack head concentrates damage in a perfectly smooth failure area. Thus, intermediate cases can exist between these extreme cases where the dominium is neither a volume nor an area. It is a fractal dominium that has dimensions between 3 and 2. We can consider this dominium as fractal geometry where the power-laws exponent, ${d}_{G}$, can vary between 0 and 1.

These fractal exponents have a range of variations depending on the geometrical shape of the specimens, the boundary condition, the characteristics of the concrete as maximum aggregate size, and quantities of its components, among others. References^3,35,36,40,41,48,49^ experimentally found that the fractal exponent of the tensile stress varies between 0.091 and 0.41, the fractal exponent of the fracture energy varies between 0.085 and 0.48, and the fractal exponent of the critical strains varies between 0.48 and 0.73.

## The lattice discrete element method formulation

**Figure 3:**
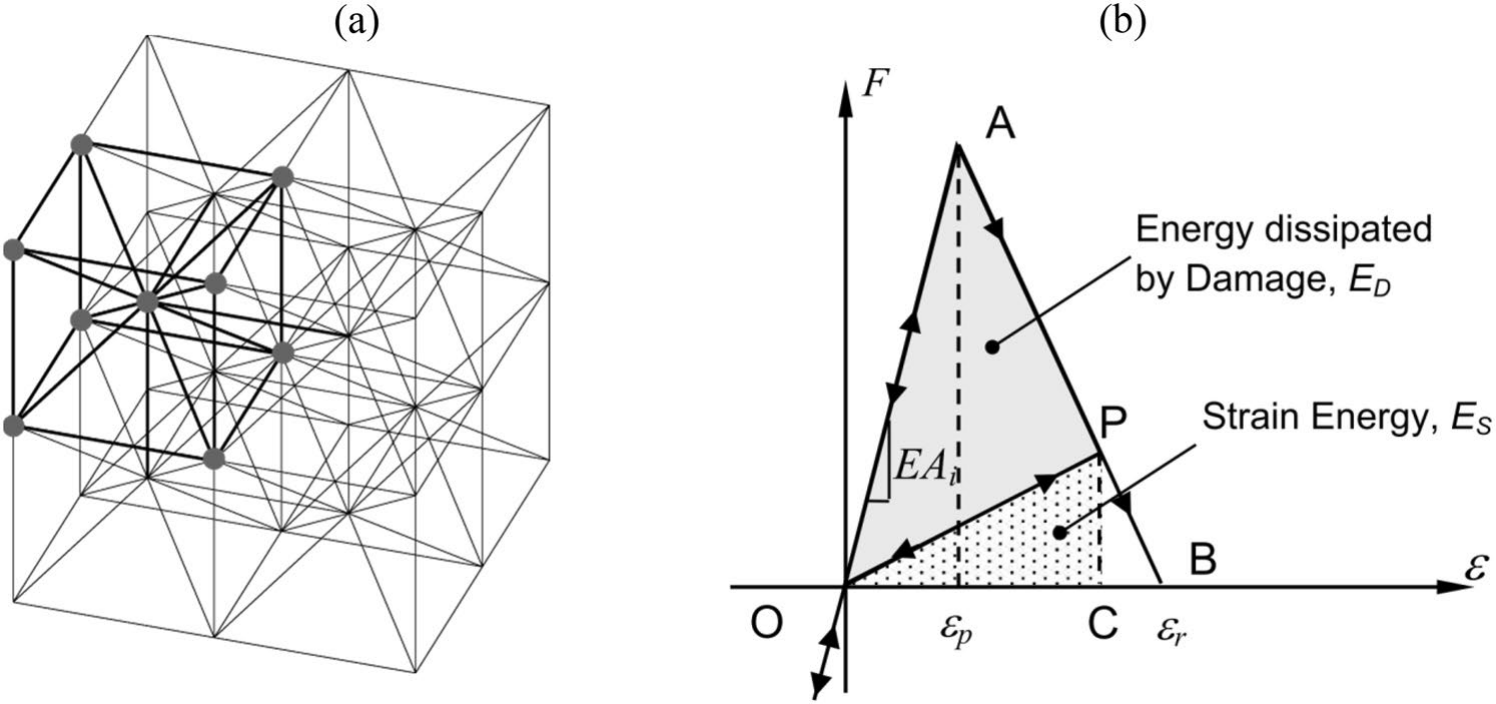
View of basic LDEM model: (**a**) cubic arrangement of nodes and massless uniaxial elements (bars), (**b**) bilinear constitutive relationship for both diagonal and longitudinal bars.

The Lattice Discrete Element Method (LDEM) models solids by an arrangement of massless uniaxial elements (bars) that can carry only axial loads. Nayfeh and Hefzy^50^ determined the properties of an orthotropic elastic continuum *equivalent* to a cubic arrangement of bars consisting of a cubic cell with nine nodes (as shown in Fig. 3a). The mass is concentrated at nodal points, each having three degrees of freedom (the displacements in the three orthogonal coordinated directions). The longitudinal and diagonal bar lengths are ${L}_{n}=L$ and ${L}_{d}=\sqrt{3}L/2$, respectively, for the basic geometric arrangement. The equations that relate the properties of the LDEM bars with the elastic *isotropic medium* parameters may be found in Ref.^51^. The cubic array considered by Nayfeh and Hefzy^50^ results in the exact representation of the isotropic continuum for $\nu =1/4$. Slight differences appear in the shear terms for other values of $\nu$. The cross-section of each diagonal and longitudinal bar, *A*_*d*_ and *A*_*n*_*,* links the stiffness of each bar to the properties of the isotropic elastic medium.

3 $${A}_{n}=\phi {L}^{2}, {A}_{d}=\frac{2}{\sqrt{3}}\delta {A}_{n}=\frac{2}{\sqrt{3}}\delta \phi {L}^{2},$$

*E* and ν are the materials’ the elastic modulus and the Poisson coefficient, respectively, and the function $\phi =\left(9+8\delta \right)/\left(18+24\delta \right)$ and $\delta =9\nu /\left(4-8\nu \right)$.

The discretized equation of motion according to Newton’s second law are obtained from equilibrium conditions of all forces acting on the nodal mass, resulting in a system of equations of the form:

4 $${\varvec{M}}\ddot{x}\left(t\right)+{\varvec{C}}\dot{x}\left(t\right)+{\varvec{F}}\left(t\right)-{\varvec{P}}\left(t\right)=0$$

where the vectors $\ddot{x}(t)$ and $\dot{x}\left(t\right)$ represent the nodal acceleration and velocity, respectively, ${\varvec{M}}$ and ${\varvec{C}}$ are the mass and damping matrices. Moreover, the vectors ${\varvec{F}}\left(t\right)$ and ${\varvec{P}}\left(t\right)$ are the internal and external nodal forces, respectively. The damping matrix ${\varvec{C}}$ is given by:

5 $${\varvec{C}}=2\pi \xi {f}_{p}{\varvec{M}}$$

where $\xi$ is the damping ratio, and ${f}_{p}$ is the frequency at the peak value of the energy spectrum. Since ${\varvec{M}}$ and ${\varvec{C}}$ are diagonal matrices, the scalar equations corresponding to the vector given by Eq. (4) are integrated into the time domain by using an explicit finite difference scheme. Note that, since nodal coordinates are updated at each time step *t*, they can directly be computed from the above equations without any additional computation, even in the case of large displacements because rotations are not degrees of freedom of the model. The convergence of LDEM solutions in linear elasticity and elastic instability problems was verified by^52^, among others.

Hilleborg’s^10^ proposal to model the behavior of quasi-brittle materials is used to represent the LDEM uniaxial bilinear constitutive relationship for longitudinal and diagonal bars. The softening branch accounts for the irreversible effects of fracture, nucleation, and propagation. The area under the force-strain curve (the area of the OAB triangle in Fig. 3b) is proportional to the energy necessary to fracture the bar, i.e., the fracture energy. Thus, considering an intermediate state during the damage process represented at the element scale by the point P on the constitutive relation, the area defined by the OAP triangle quantifies the energy dissipated by the damage. When the damage energy is equal to the area of OAB triangle, the bar subjected to tension loses its load carrying capacity.

On the other hand, under compression, the material behaves in a linear elastic manner. Thus, failure in compression will be non-local and induced by indirect traction. This assumption is reasonable in quasi-brittle materials because its ultimate strength in compression is usually five to ten times larger than in tension^34^.

From the bilinear constitutive relationship, when ε is equal to or greater than ε_*r*_ (Fig. 3b), the critical condition of the member is reached. The critical strain of each element, ε_*r*_, is equal to:

6 $${\varepsilon }_{r}={\varepsilon }_{p}{d}_{eq}\left(\frac{{A}_{i}^{*}}{{A}_{i}}\right)\left(\frac{2}{{L}_{i}}\right),$$

where ε_*p*_ is the strain at the peak load, and *d*_*eq*_ is a characteristic length of the material (similar to the width of the plasticity region in the crack tip in the Dugdale model). In Eq. (6), *i* identifies the bar type (*i* = *d* for a diagonal bar, and *i* = *n* for a longitudinal bar), *L*_*i*_ is the bar length, *A*_*i*_ is the bar cross-section, and finally ${A}_{i}^{*}$ is the equivalent fracture area of the *i*-th bar.

As showen in Refs.^33,51,53^ the equivalent fracture area ${A}_{n}^{*}$ can be deduced by equating the dissipated energy ($\Gamma$) in a cubic continuum specimen with dimensions *L* × *L* × *L,* because of a fracture along a plane parallel to one of its faces

7 $$\Gamma ={G}_{f}{L}^{2}$$

to the dissipated energy in a LDEM cell (${\Gamma }_{LDEM}$) of sizes *L* × *L* × *L* along the same fracture plane

8 $${\Gamma }_{LDEM}={G}_{f}\left[4\left(\frac{1}{4}\right){A}_{n}^{*}+{A}_{n}^{*}+4\left(\frac{2}{\sqrt{3}}\delta {A}_{n}^{*}\right)\right]{L}^{2}$$

where ${G}_{f}$ is the fracture energy related to the size *L*. The first term within square brackets of Eq. (8) accounts for the contribution of the four external longitudinal bars of the LDEM cell (each of them sheared with the other four adjacent cells, as is shown Fig. 7a). The second term accounts for the internal longitudinal bar (the vertical one in Fig. 7a). The third term within square brackets of Eq. (8) accounts for the four diagonal bars, where the factor $2/\sqrt{3}\delta$ is the ratio between the diagonal and the longitudinal bars. This factor, the quotient between Expressions (3), is a property of this cubic arrangement. Therefore, by equating Eq. (7) with Eq. (8), ${A}_{n}^{*}$ is obtained and consequently ${A}_{d}^{*}$ as follows:

9 $${A}_{n}^{*}\cong 0.138963{L}^{2}\cdots {A}_{d}^{*}=\frac{2}{\sqrt{3}}\delta {A}_{n}^{*}\cong 0.1805{L}^{2}\cong 0.135389{{L}_{d}}^{2}$$

Coming back to the critical strain definition (Eq. 6), the bar is considered as “broken” when its strain is equal to or greater than the critical strain value, ${\varepsilon }_{r}$. Note that the bar behavior under compression is assumed linearly elastic. A compressive failure in a body can occur due to the transversal tension stresses produced by the axial compression because failure under compression is not allowed on the element.

The strain at the peak load, ε_*p*_, is computed as follows:

10 $${\varepsilon }_{p}=\sqrt{\frac{{\mu G}_{f}}{E{d}_{eq}}}$$

In Eq. (10), ε_*p*_ denotes the strain at the peak of the stress vs. strain law, *µG*_*f*_ is the mean fracture energy of the material, *E* is the Young's modulus, and finally *d*_*eq*_ the characteristic material length which is also considered a material property. The unstable fracture propagation, a situation considered and studied in the following section, requires that the characteristic length of the structure exceeds *d*_*eq*_. The role of the material characteristic length in the fracture process is discussed also by^54^.

In the LDEM, *G*_*f*_ is assumed to have a probability distribution proposed by Weibull^4^ given by:

11 $$p\left({G}_{f}\right)=1-exp[-{({G}_{f}/\beta )}^{\gamma }]$$

As explained in Ref.^4^, *G*_*f*_ is associated with the statistical distribution of the weakest portion of the system tested. Notice also that the Weibull distribution interval is [0, infinity], which is very convenient to simulate values of *G*_*f*_ where nonphysical sense will have negative values. Hansen^55^ presents an excellent explanation of the extremal statistic distribution and their application. In Eq. (11), *β* and *γ* are the scale and shape parameters, respectively. They can be computed through the coefficient of variation ${CV}_{{G}_{f}}$ (defined as the ratio between the standard deviation) and the mean value ${\mu G}_{f}$ of the specific fracture energy *G*_*f*_ related to the size *L*. However, a spatial correlation function for *G*_*f*_ needs to be defined. The spatial correlation for *G*_*f*_ random field describe the dependence among the value specified in spatial coordinates and their neighbors' values. The correlation lengths *L*_*cx*_, *L*_*cy*_, and *L*_*cz*_, along with the three directions *x*, *y*, and *z*, respectively, are used to achieve such purpose^56^. In the proposal, the random field domain is discretized by a regular prismatic arrangement of poles with size defined by *L*_*cx*_, *L*_*cy*_, and *L*_*cz*_. An uncorrelated value, *G*_*f*_ (X_pk_, Y_pk_, Z_pk_) is associated with each pole k being X_pk_, Y_pk_, Z_pk_ its spatial coordinates. The correlated value of the i-th bar *G*_*f*_ (x_i_, y_i_, z_i_) is assigned at each bar with barycentric coordinates x_i_, y_i_, z_i_. This value is computed by linear interpolation of the eight *G*_*f*_ values attributed to the poles that define the prismatic domain where the barycentric bar is. Details may be found in Refs.^57,58^. It is important to emphasize that this approach to considering the random spatial distribution is straightforward. In the specialized literature, this topic is described more accurately in the books of Ziman^59^ and Ostoja-Starzewski^60^. On the other hand, in Demmie and Ostoja-Starzewski^61^, the authors applied a deep study focusing on the sensibility of the spatial distribution of the random material properties in the simulation result. The cited study uses a known version of the discrete element strategy based on Perydinamic.

**Figure 4:**
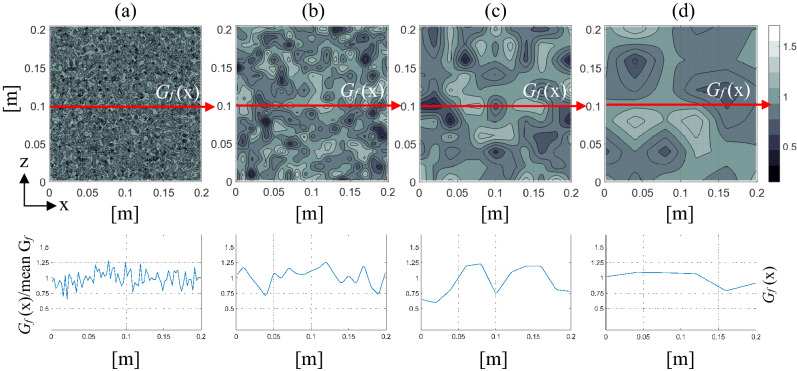
Distribution of normalized *G*_*f*_ : using *L*_*cx*_ = *L*_*cz*_ equal to (**a**) 0.15 cm; (**b**) 1 cm; (**c**) 2 cm and (**d**) 4 cm with the element size *L* = 0.005 m.

To exemplify the influence of the random field produced with *G*_*f*_ (which is assumed to have a Weibull probability distribution presented in Eq. (11) through the correlation length), a square plate with a side of 0.2 m is analyzed in plane strain condition. Figure 4 shows the normalized distribution of *G*_*f*_ referring to the mean *G*_*f*_ value, corresponding to four pairs of values of the correlation lengths with *L*_*cx*_ = *L*_*cz*_. This example, similar to the one found in Ref.^57^, shows that the *G*_*f*_ profile is susceptible to the spatial correlation lengths and that they can considerably affect the crack path.

## Application: rock specimens with different size submitted to uniaxial tensile stress

The failure of square rock specimens with side *b* ranging from 0.05 to 3.50 m is simulated in this work to introduce the cohesive fractal law in the LDEM environment. Reference^34^ uses this same group rock specimen to study the size effect and the ductile–brittle transition. The samples have their lower face fixed, while their upper faces have displacements that increase monotonically (with a strain rate of 0.1/s). In all cases, we restrict the nodal displacements in the thickness direction to achieve plane strain conditions. In the subsequent research could be interesting to verify the interaction between the scale effect and the strain rate in the context of LDEM. Preliminary studies of this interaction were made by Refs.^62,63^.

The smallest LDEM array that leads to satisfactory results consists of 10 × 10 × 1 cubic modules with 1026 degrees of freedom (used for the smallest model *b* = 0.05 m). The largest specimen used in this study is the 3.50 m side model. It consists of 700 × 700 × 1 cubic modules with 1,472,802 degrees of freedom. Table 2 shows the basic dimensions of the samples.

**Table 2:** The dimensions of the LDEM models studied.

Specimen	1	2	3	4	5	6	7	8	9	10	11	12	13
*b* (m)	0.05	0.075	0.10	0.15	0.20	0.25	0.30	0.40	0.50	0.75	1.00	1.50	3.50
Cells	10	15	20	30	40	50	60	80	100	150	200	300	700

Table 3 indicates the relevant material properties. It is important to note that the fracture energy *G*_*f*_ related to the size *L* is modeled as a 3D random field. That is to say, the probability distribution of *G*_*f*_ was assumed as a Weibull function with mean value, coefficient of variation, and correlation length described in Table 3. The distance where no correlation among energy values can be found in this random field is known as correlation length.

**Table 3:** Relevant rock (granite) material properties and LDEM parameters.

Material properties	Value
*E* (Young’s modulus)	75 GPa
*ρ* (specific mass)	2700 kg/m^3^
*ν* (Poisson coefficient)	0.25
*d*_*eq*_	1.465 m
μ*G*_*f*_ (Mean fracture energy related to size *L*)	1300 N/m
CV(*G*_*f*_) (coefficient of variation of *G*_*f*_ )	40%
Correlation length of *G*_*f*_ random field	0.0015 m
*L* (basic modulus length)	0.005 m

Each simulation leads to different stress–strain curves and strengths as the material properties are associated with a statistical distribution. For this reason, four simulations were carried out for each size specimen to obtain representative results.

As presented in Table 3, the correlation length adopted in the first application is *L*_*cx*_ = *L*_*cz*_ = 1.5 mm, which is related to the material microstructure. The correlation length used in this work is smaller than the LDEM basic module length. For this reason, the random values of *G*_*f*_ assigned to every bar are statistically independent. That is, the properties of one bar do not depend on the properties of the neighboring/adjacent ones.

**Figure 5:**
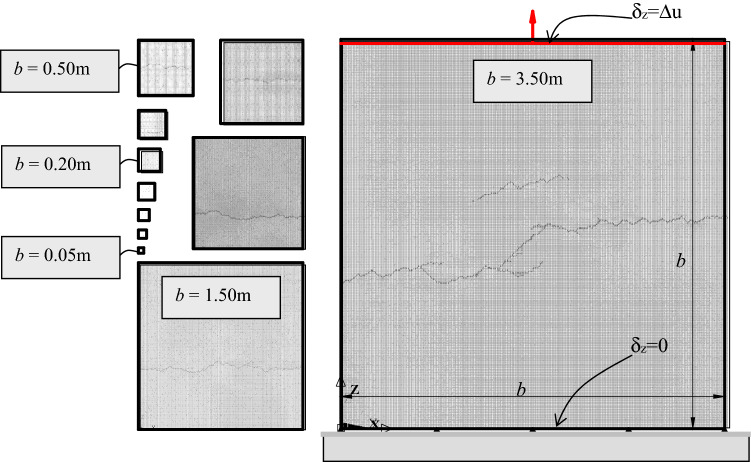
The relative size of the specimens and boundary conditions considered. The damage distribution and failure configuration of specimens of various sizes subjected to applied displacements inducing uniaxial tension. The characteristic specimen size b varies between 0.05 and 3.5 m.

One representative sample of the final simulated configuration is shown in Fig. 5, in which the colors light gray, gray, and black represent undamaged, damaged, and broken (failed) elements, respectively.

**Figure 6:**
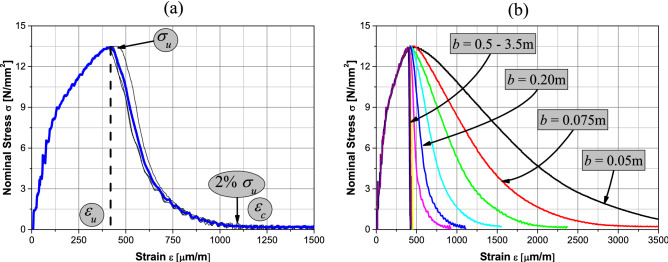
(**a**) Curves for the mean vertical stress at lower support vs. mean strain for the b = 0.20 m rock specimen obtained in four simulations and average curve (in blue), and (**b**) mean stress–strain curves for the different size specimens.

Figure 6a presents the resulting stress–strain curves, and the average one, for all simulations of the *b* = 0.20 m specimen. The main parameters that characterize the stress–strain curves are represented in Fig. 6a: *σ*_*u*_ denotes the ultimate stress, *ε*_*u*_ the strain related to the ultimate stress, and *ε*_*c*_ the critical strain or the strain at the point where the strength is exhausted. For practical purposes, the critical strain *ε*_*c*_ is defined as the strain where the stress decreases below 2% of the maximum stress (*σ*_*u*_). This notation is applicable, without any restriction, to specimens with sides smaller than 0.4 m. For specimens of lengths equal to 0.4 m or larger, failure occurs in a brittle manner and the critical strain *ε*_*c*_ cannot be distinguished from the strain at peak *ε*_*u*_.

Figure 6b shows the mean curves for all specimen sizes. It may be seen that the shape of the mean stress–strain curves varies with the specimen size. This feature has been repeatedly observed in experimental studies such as Refs.^48,49^. In the context of the numerical method used here, Birck et al*.*^64^ analyzes the relation between the global response's curve shape and the specimens' size. It is important to note that only self-similar configurations were utilized. There are cases in which the numerical simulation produces different failure patterns, sometimes with only one crack and others with two or more. All the studies carried out next always utilized simulations with only one crack propagation. Figure 6b shows that the curve falls after reaching the maximum strength for all specimens size b [0.5–3.5 m]. It is possible to avoid this behavior carried out a load control during the test as made by Refs.^48,49^. Simulating this behavior to capture the unstable branch in the global stress–strain curve using LDEM will be the topic of future work of our research group.

As shown in Fig. 6a, it is possible to specify the stress-stain curve by identifying some characteristic values without losing essential information. Table 4 lists the corresponding mean characteristic values of the strain–stress curve to the specimens’ simulations with different sizes.

**Table 4:** Mean values of stress and strain at peak load and failure strain to each of the specimen sizes studied.

*b* [mm]	*σ*_*u*_ [N/mm^2^]	CV [%]	*ε*_*u*_ [μm/m]	CV [%]	*ε*_*c*_ [μm/m]	CV [%]
50	13.461	1.10	523	7.42	4509	4.53
75	13.518	0.85	456	7.30	2807	5.01
100	13.415	0.98	433	5.63	2073	4.08
150	13.508	0.63	428	0.04	1418	0.14
200	13.473	0.39	424	3.13	1067	2.44
250	13.429	0.68	421	1.31	812	1.76
300	13.393	0.16	412	0.15	615	4.77
400	13.488	0.31	414	1.29	460	3.33
500	13.471	0.30	410	1.36	457	2.41
750	13.455	0.39	402	1.92	429	1.72
1000	13.420	0.20	397	1.24	420	1.20
1500	13.437	0.20	403	0.76	430	0.76
3500	13.347	0.21	379	1.87	413	0.60

Kosteski et al.^34^ analyzes the Carpinteri’s brittleness number *s* for this same set of specimens. The definition of this dimensionless number allows characterizing the structural response as brittle, ductile, or brittle-ductile transition behavior. This work verified the concordance between the results obtained with LDEM simulation and the experimental ones obtained from the technical literature.

## Cohesive fractal law of numerical simulations

It is possible to measure the fractal dimensions of the simulations presented in the previous section as defined by Carpinteri^35^. It is made in this section.

### Fractal tensile strength ${{\varvec{\sigma}}}^{\boldsymbol{*}}$

The fractal exponent of tensile strength is the slope of the bi-logarithmic normal strength versus characteristic specimen size curve, that is, a measure of the tensile scale effect. When *d*_*σ*_ = 0, there is no scale effect on tensile strength, the bigger *d*_*σ*_, the bigger the scale effect will be.

**Figure 7:**
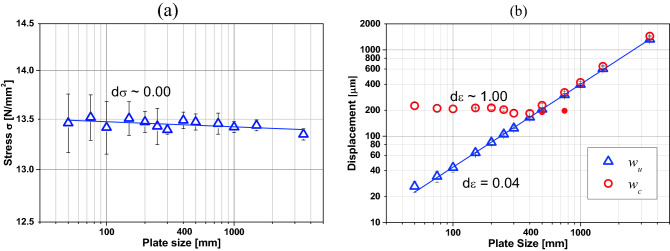
(**a**) Ultimate global stress. (**b**) Ultimate and characteristic global displacement versus the specimen dimension. The mean values and bar with ± 2 standard deviation are indicated in the figure.

Figure 7a shows the bi-logarithmic mean strength versus specimen size curve, where a tendency line obtained by minimal squares is also presented. This line’s slope and the constant are the fractal exponent and the fractal tensile strength ${\sigma }_{u}^{*}$, respectively. In addition, the fractal tensile strength is a true material scale-invariant. Therefore, the practical null scale effect was found in tensile stresses *d*_*σ*_ = 0.002, and ${\sigma }_{u}^{*}=13.568$ MN/mm^1.998^. Figure 7a shows also that the variability of the values is considerable in the case of the small specimens, and after *b* = 0.2 m, this variation diminishes.

### Fractal critical strain ${{\varvec{\varepsilon}}}^{\boldsymbol{*}}$

Following similar reasoning as the previous one, it is possible to find the fractal exponent of deformations *d*_*ε*_ measuring a slope of the bi-logarithmic critical displacement (remembering that *w*_*c*_ = *ε*_*c*_* b*) versus characteristic specimen size curve (*b*).

Figure 7b shows, in a bi-logarithmic scale, the relations between *w*_*u*_ and *w*_*c*_ with the specimen size *b*. It may be seen that *w*_*c*_ presents two typical regions: for specimens larger than 0.4 m, it decreases with the specimen size. However, specimens smaller than about 0.4 m remain practically invariant, at least within the range of sizes and material properties herein examined. This behavior is clearly illustrated by *s* value, which shows that the largest specimens fail in a brittle manner (see^34^). For the larger specimens (*b *$\ge$ 0.4 m), the correct value of final strain *ε*_*c*_ must be lower than *ε*_*u*_, thus, failure becomes unstable.

Therefore, when fitting a line in the bi-logarithmic graphic of Fig. 7b for b < 0.2 m specimens, the fractal exponent and the fractal strain are found for the peak displacements ${\varepsilon }_{u}^{*}=526$ μm/m and *dε*_*u*_ = 0.04, and for the critical displacements ${\varepsilon }_{c}^{*}=210$ μm/m and *dε*_*c*_  $\cong \hspace{0.17em}$ 1. In the case of specimens bigger than 0.40 m, the fractal exponent, for both the peak and critical displacements, is *dε* $\cong$ 0.04, and the fractal strains are ${\varepsilon }_{u}^{*}=526$ μm/m and ${\varepsilon }_{c}^{*}=575$ μm/m, values relatively close to each other. In the case of bigger specimens, the peak and the critical strains get confused. That is, there is no significant difference between these points. However, there is no crack location before the failure. For this reason, the fractal exponent is small, near zero. As a result, the specimens broke abruptly, so they were unstable.

It is possible to see that the fractal stain exponent obtained with the peak displacements (displacement related to the maximum stress) is practically null in specimens smaller than 0.2 m, which shows a ductile behavior. That is to say, at this point, there is no strain localization. However, when calculating the fractal exponent with the critical strain (2% of the maximums stress), a unitary value is obtained as a result. This means that the crack has already been localized. Therefore, the fractal exponent goes from 0 to 1, depending on where the strain is considered.

**Figure 8:**
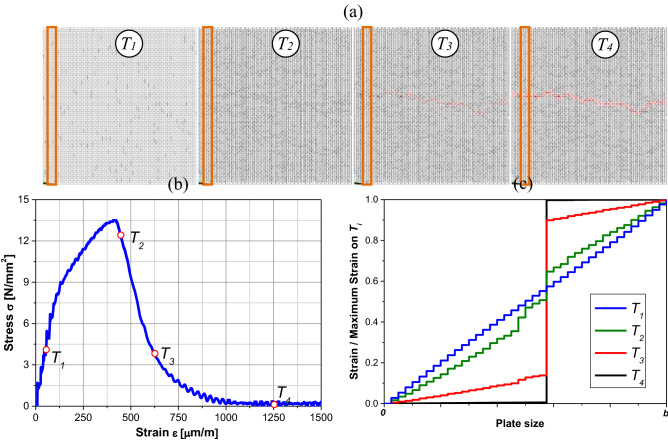
(**a**) Configuration of a specimen size *b* = 0.2 m at four different times, (**b**) points on stress–strain curve defining the time of the configurations, (**c**) normalized strains for the strips highlighted on (**a**).

The configurations presented in Fig. 8a were studied to explain this phenomenon better. This Figure shows the configuration of one specimen with size *b* = 0.20 m at four different times, which are shown on the strain stress curve of Fig. 8b. The damaged elements are represented in Fig. 8a in black and the broken ones in red. As shown in Fig. 8a, the first image, T_1_, happens before the maximum stress, and it is possible to see the distributed damage through the specimen. The second image is obtained after the maximum stress point, and it is possible to see broken elements, that is to say, that the damage nucleation has already taken place. In the third image of Fig. 8a, T_3_, shows the specimen with a practically defined crack, while in the last image, T_4_, the specimen broken with a well-defined crack is observed.

Figure 8c shows the normalized displacement of the strip highlighted in Fig. 8a at each point showed in Fig. 8b. It is important to note that the deformations are concentrated on the LDEM node mesh. This figure is compatible with Fig. 1, which shows the extremely diffused strain (*d*_*ε*_ = 0) and the extremely localized deformation (*d*_*ε*_ = 1) over a bar. Therefore, it is noted that along the failure process, the specimen strip analyzed changes its fractal exponent from 0 to 1 since the deformations return to zero in the regions outside the crack (the LDEM constitutive law is linearly elastic and the discharge goes to the origin). Hereby, the value of the fractal strain exponent is related before starting the damage nucleation.

### Fractal fracture energy ${G}_{f}^{*}$

**Figure 9:**
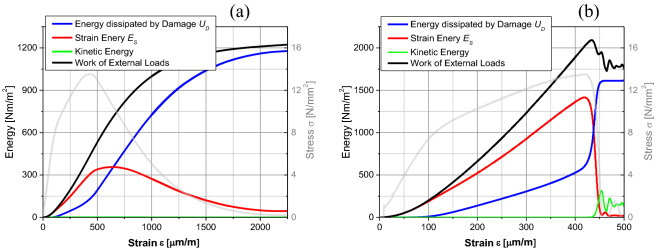
Variation of the strain energy and the energy dissipated by damage per unit area (divided by the cross-section of the specimen, *b t*) of one representative specimen with size (**a**) *b* = 0.10 m and (**b**) *b* = 0.5 m.

Figure 9 shows the variation of strain energy and energy dissipated by damage per unit area (divided by the specimen's cross-section, *b t*) of one representative specimen with sizes *b* = 0.10 m and *b* = 0.5 m. This figure shows the difference between a specimen with stable propagation and an unstable one. In the first case, the kinetic energy is disregarded in the entire process because it is negligible related to the energy dissipated by damage or the strain energy, as shown in Table 5. It is important to note that the stress peak does not necessarily coincide with the maximum strain energy. However, it is at this last point that the localization process starts. Notice in Fig. 9b that, after the peak of strength to reach, the kinetic energy continues to have a finite value due to the rigid motion of the upper portion of the broken specimen.

**Table 5:** Mean values of the kinetic energy *E*_*K*_, the strain energy *E*_*S*_ and the energy dissipated by damage *U*_*D*_, referred to the peak of stresses and to the final failure process (2% of the maximum stress) divided by the cross section of the specimen (*b t*).

*b* [mm]	At peak energy [Nm/m^2^]						At ε_c_ (2% σ_u_) energy [Nm/m^2^]						Released energy (7)
	*E*_*K*_ (1)	*CV* (%)	*E*_*S*_ (2)	*CV* (%)	*U*_*D*_ (3)	*CV* (%)	*E*_*K*_ (4)	*CV* (%)	*E*_*S*_ (5)	*CV* (%)	*U*_*D*_ (6)	*CV* (%)	*U*_*total*_–*U*_*Dpeak*_
50	0.8	56	161.0	5.1	74.8	10.9	0.2	51	21.0	14	1098.3	6.3	1044.5
75	0.8	48	231.5	5.0	103.8	9.7	0.1	53	25.0	8.7	1150.7	4.2	1071.9
100	0.9	40	289.6	4.9	125.7	8.5	0.2	56	16.9	2.9	1111.0	2.1	1002.2
150	0.9	40	435.3	4.6	183.5	8.4	0.3	45	23.8	5.7	1217.3	2.7	1057.6
200	1.0	35	561.4	3.9	229.4	6.8	0.6	41	24.1	12	1222.8	4.3	1017.5
250	1.0	30	682.2	4.2	272.3	8.0	1.4	144	25.0	56	1259.8	2.8	1012.5
300	0.9	36	829.8	3.8	351.8	6.4	11.5	47	23.7	50	1365.5	8.7	1037.4
400	1.0	33	1138.2	3.0	494.3	5.8	99.9	55	82.2	42	1705.3	6.5	1293.2
500	1.5	35	1375.2	2.8	545.4	6.4	257.2	51	185.9	49	1683.5	5.4	1324.0
750	2.2	17	2018.5	2.7	792.6	5.4	460.3	67	542.2	32	2011.7	10.4	1761.3
1000	3.4	89	2617.7	1.6	1017.1	3.0	508.9	36	635.5	41	2689.3	12.0	2307.7
1500	10.5	38	4018.4	2.8	1608.4	4.5	1279.3	27	1149.0	42	3538.3	13.5	3078.9
3500	16.2	32	8834.7	2.5	3237.5	5.1	3435.2	20	2091.7	53	7704.1	12.9	6558.3

When the specimens present an unstable propagation, the kinetic energy is also neglected at the peak of stresses, which in these cases, the peak coincides with the maximum of the strain energy. When the crack is localized in these specimens, the unstable propagation starts and the kinetic energy shoots up at a value that depends on where the crack is located. The upper part of the specimen continues to be dislocated and the inferior one back to its undeformed position. The elastic waves generated during the crack process and the kinetic energy play an essential role at this moment, but they are very dependent on the place of the crack or the sizes of the two broken parts. For this reason, the variability of final kinetic and strain energies is high (see Table 5) for the specimens with unstable propagation.

Table 5 presents the mean values of the energies obtained during the simulation process divided by the specimen’s cross-section (*b t*). They are the kinetic energy *E*_*K*_, the strain energy *E*_*S*_ and the energy dissipated by damage *U*_*D*_, referring to the peak of stresses and to the final failure process (2% of the maximum stress).

**Figure 10:**
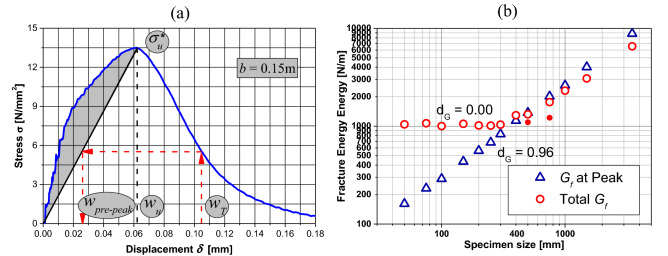
(**a**) Stress-displacement curve for *b* = 0.15 m showing various displacement definitions. (**b**) Fracture energy versus the specimen dimension. The mean values and bar with ± 2 standard deviation are indicated in the figure.

In the LDEM simulations, it is considered that the discharge (or unload) is always directed to the origin (as shown in Fig. 10a), following a damaged elastic modulus instead of the initial slope of the curve as defined in Fig. 2. Another important issue is that the stress displacement curve area is related to the external work done by the system. This energy must be equal to the internal energy balance. According to that, the fracture energy *G*_*f*_ can be found both by measuring the area of the stress-displacement curve or by energy balance. This definition is only valid for the specimens with a stable propagation, i.e., those with a “ductile” behavior. According to this last option, *G*_*f*_ is the total energy at the end of the fracture process minus the energy dissipated by damage (*U*_*D*_) referred to the peak of stresses (related to the marked area in Fig. 10a).

The value of the released energy shown in Table 5 (column 7) is calculated as defined earlier by the sum of the strain energy (column 5) plus the energy dissipated by damage (column 6) at the end of the failure process minus the energy dissipated by damage (column 3) at the peak of stresses. As was explained before, this released energy coincides with the fracture energy when the specimens have stable crack propagation. Following this idea, the strain energy by unit area at the peak of the stresses (column 2) would be the “fracture energy” or the energy released before the damage nucleation occurs. When the specimens present an unstable crack propagation, column 7 of Table 5 represents the sum of both the emitted and the dissipated energies.

Figure 10b shows the fracture energy variation with the specimen size at the end of the failure process and at the maximum stress on the bi-logarithmic scale. Therefore, from Fig. 10b, when the energies at final damage are used, the slope of the curve is around zero for specimens with ductile or brittle-ductile transition behavior (${G}_{f}^{*}\approx 1030$ Nm/m^2^). When the energies related to the peak of stresses are used, the slope of the curve is *d*_*G*_ = 0.96 for all the specimen sizes, and the fractal fracture energy is ${G}_{f}^{*}\approx 3.58$ Nm/m^2.96^.

It is interesting to note that when analyzing the energies, issues are found that are similar to those dealt with when analyzing the strains or displacements. When the variables are related to the stress peak, there is no localization because the energy dissipates by the specimen volume with *d*_*G*_ near 1. When there is localization, the fracture energy is spent along the surface crack, and *d*_*G*_ tends to be 0. For the bigger specimens whit a brittle failure, the propagation is fast (see the kinetic energy), and more places on them that are damaged. Then the energy dissipates in a dimension smaller than the specimen volume but bigger than a surface.

### Cohesive fractal law: relation between the fractal exponents

Table 6 present a summary of all the values of fractal exponent obtained by LDEM simulation in the previous sections. The values obtained for the specimens with stable crack propagation are considered the values that describe the behavior of all specimens. Carpinteri’s cohesive fractal law establishes that the sum of three fractal exponents must equal 1. This is observed in the LDEM simulations.

**Table 6:** Summary of LDEM simulation fractal exponents.

*d*_*σ*_	*d*_*ε*_		*d*_*G*_	
	At peak stress	At failure	At peak stress	At failure
0.00	0.04	1.00	0.96	0.00

**Figure 11:**
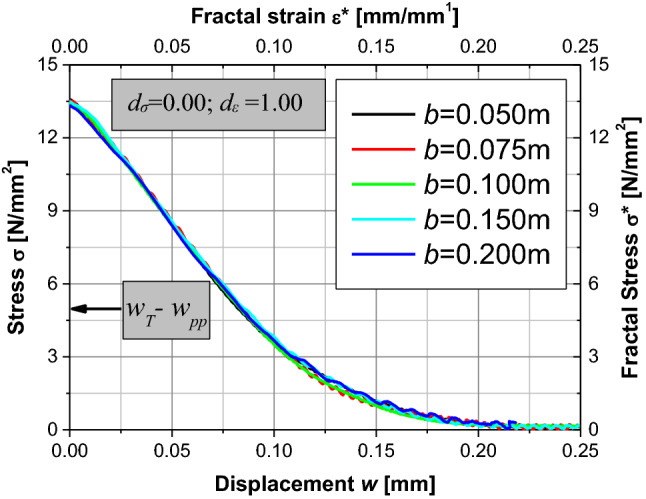
Stress displacement curve that for these fractal exponents coincide with the fractal stress–strain curve.

The stress-displacement curves for specimens with ductile behavior, *b* < 0.2 m, shows better the softening branch after the peak of stresses. Therefore, these curves will be used to find the cohesive fractal law and the fractal exponent for this specimen’s interval. Thereby, as shown in Table 6, the fractal exponent that will be used are *d*_*σ*_ = 0.00, *d*_*ε*_ = 1.00 e *d*_*G*_ = 0.00. This means there is no difference between the stress versus *w*_*T*_* –w*_*pp*_ curve and the fractal one (see Table 1 for *d*_*ε*_ = 1). In this way, Fig. 11 also shows the fractal stress (${\sigma }^{*}=\sigma {b}^{{d}_{\sigma }}$) versus the fractal strain (${\varepsilon }^{*}$) curve obtained for these specimens for *ε* ≥ *ε*_c_. In the fractal dominium, this relation is independent of size.

It is important to note that practically no scale effect is found for the rock properties utilized in these simulations. Nevertheless, the authors decided to show these results, which were also used in previous works as^34^, to describe the methodology and process of obtaining the fractal dimensions as invariant. Item 6 will show how the LDEM method can get the fractal dimension closest to the one obtained by other researchers for concrete materials and the fractal stress–strain curve obtained for these cases.

## Sensibility study of the cohesive fractal law

Several experimental studies in which the fractal exponent of concrete or rocks are measured (see Refs.^3,35,36,40,41,48,49^) found that the tensile fracture exponent varies between 0.09 and 0.41. These studies also found that the fractals critical strain varies between 0.48 and 0.73, and finally, the fractal exponent of the fracture energy varies between 0.085 and 0.48. These values are far from the ones obtained in the previous sections. As it was explained earlier, this example was adopted to explain in detail every point of the fractal independent size cohesive law. To show that the LDEM simulation can correctly represent the physical size effect problem for quasi-brittle materials, in this section are shown the fractal parameters found when some parameters are modified. The three main parameters here analyzed are the following: the correlation length of the random field, the adopted constitutive law, and finally, the boundary condition of the problem. Each of these parameters changes the fractal exponent results and can have a coupled effect on them.

### Influence of the correlation length of the random field

As showed in Puglia et al*.*^57^, the correlation length of the *G*_*f*_ random field is responsible for the objectivity of the LDEM mesh. When a material is defined, and its properties remain constant with the change of the LDEM mesh**,** the results do not change. In the LDEM simulation, it is assumed that the fracture energy varies spatially in a model with a defined mean value and a Type III Weibull distribution. The correlation length, i.e., the length at which the properties are not correlated anymore, is a material property. In Junges et al*.*^65^, it was found that the correlation length measured for a self-compacting concrete with a maximum size of the coarse aggregate of 9.5 mm is around 5 cm. This value was obtained by using both sclerometric and macro indentation and at the same time by also assuming that the fracture energy has the same spatial distribution that the material hardness.

It is important to note that this correlation length is a parameter that no one commonly measures or reports in their works. In the rock simulations presented in the previous section and in Kosteski et al.^34^, the correlation length utilized is 1.5 mm, a value which is possible to find in small grain hard rock, but difficult to find in concretes. The same samples presented in “Application: rock specimens with different size submitted to uniaxial tensile stress” were simulated with the same properties but changing the fracture energy correlation length, *L*_*cx*_ = *L*_*cz*_ equal to 0.15 cm, 1 cm, 2 cm, and 4 cm, to analyze the influence of this parameter. Figure 4 shows the spatial distribution of normalized fracture energy of one specimen with *b* = 0.2 m for each correlation length utilized. The fracture energy is normalized by its mean value *μG*_*f*_ = 1300 N/m. Figure 4 also shows the linear cut in these specimens and the variation of the normalized fracture energy on this line. It is possible to see that when the specimen size is proximal to the correlation length utilized, the variation of this property is smoother. On the other hand, when the correlation length is much smaller than the specimen size, there are large swings of fracture energy.

It is worth noting that self-similar results with one crack propagation are also considered. When the ratio between the size specimen and the correlation length is small, i.e., small specimens and big correlation length, it is difficult to find only one crack propagation or breaks far from the borders where there are concentrations due to the boundary conditions. This same problem also arises in homogeneous or practically homogeneous specimens.

**Figure 12:**
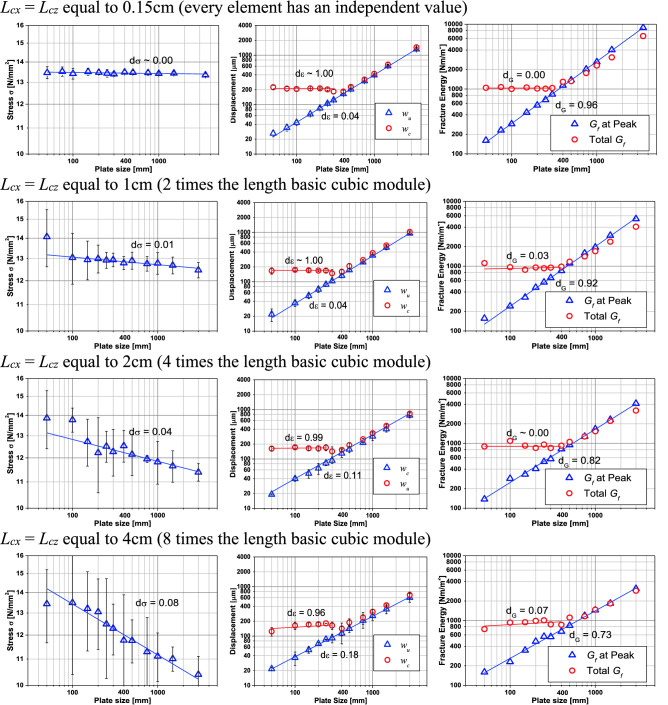
Ultimate global stress, ultimate and characteristic global displacement and fracture energy versus the specimen dimension for every size of correlation length analyzed.

Figure 12 shows, in a bi-logarithmic scale, the tensile strength, displacements, and fracture energy versus the specimen size for every correlation length being analyzed. In this figure, it is possible to see every fractal exponent calculated in the same manner as in “Cohesive fractal law of numerical simulations”. It is possible to note that the fractal exponent of tensile strength increases with the correlation length. This same behavior presents the fractal exponent of strains. The fractal exponent of fracture energy decreases with the correlation length utilized in the simulation. It is also interesting to observe that the sum of the three fractal exponents is approximately 1, as the fractal cohesive law theory describes.

In Fig. 12, it is possible to see that the variations of the results increase with the correlation length, and that the smaller specimen do not always produces good results. This happens because the mesh discretization is not so good. The specimen is modeled with only 10 × 10 LDEM basic modules. Another important issue in this analysis is that the value of mean *G*_*f*_ utilized in all the simulation is the same: a scale-invariant material parameter. This value of fracture energy utilized as input in LDEM simulations is related to the fractal fracture energy *G*_f_^*^.

If we considered linear elastic homogeneous ideals specimens with different sizes, all of them would reach the same maximum stress independent of their size. The stress–strain curve will be the same. Then, the fractal stress exponent must be zero, and the fractal strain exponent must be 1. As the strain stress curve is constant, the ε_c_ is also constant. Then, the displacement versus the size specimen will have a slope equal to 1, which indicates that the fractal exponent is equal to zero. It is worth noting that as the material is homogeneous, there will be an extremely diffused strain in all the specimens, and the failure will reach all the specimen's points simultaneously. Then if we measure the fractal strain exponent before the failure**,** we will have a *d*_*ε*_ = 0 (extremely diffused strain) and *d*_*G*_ = 1 (energy distributed along all the specimens). The fractal strain exponent is always 0 before or after the peak or failure.

If we introduce one crack in these homogeneous specimens with the same shape aspect, the maximum stress found will change with the specimen size following the LEFM, that is, *d*_*σ*_ = 0.5. As the specimen’s behavior is linearly elastic, the strain at the failure in these specimens will have a fractal exponent of *d*_*ε*_ = 0.5 (and *d*_*G*_ = 0). It is also important to note that before the failure, the stains are localized at the crack tip, then the strain must have a localized exponent, *d*_*ε*_ = 1. In this same way, the fracture energy is localized. Therefore, its exponent must be 0 (energy concentrated on one plane). This is a constant for all the specimens’ sizes as defined in the LEFM, then *d*_*G*_ = 0, after and before the failure.

In these two extreme cases, it is possible to see well-defined behaviors just before the failure and at the failure when propagation starts. The fractal strain exponent goes from *d*_*ε*_ = 1 to *d*_*ε*_ = 0.5 for homogeneous specimens with a single crack, while it remains constant (*d*_*ε*_ = 0) for homogeneous specimens without any crack. When the fractal strain exponent changes with the evolution of the failure process, the energy fracture exponent remains constant.

Table 7 summarizes the fractal exponent found in the Fig. 12 simulations. It is possible to see that the sum of three fractal exponents is approximately equal to 1 in all the cases analyzed at peak stress. This makes sense since the stress fractal exponent is obtained from the maximum stress (peak stress), the fractal exponent of strain at the peak is a description of the strain distribution, and the fractal fracture energy exponent also represents the distribution of the damage along the specimen and the stress peak. These fractal values are related to the microstructure of the specimen. When we have a more considerable heterogeneity (*L*_*c*_ smaller related to the size specimen), there is more variability in the properties, and the strain tends to be diffused. At the same time, the fractal fracture energy releases practically in the entire specimen.

**Table 7:** Summary of LDEM simulation fractal exponents.

*L*_*cx*_ = *L*_*cz*_	*d*_*σ*_	*d*_*ε*_		*d*_*G*_	
		At peak stress	At failure	At peak stress	At failure
Homogeneous	0	0		1	
0.15 cm	0.00	0.04	1.00	0.96	0.00
1 cm	0.01	0.04	1.00	0.92	0.03
2 cm	0.04	0.11	0.99	0.82	0.00
4 cm	0.08	0.18	0.91	0.73	0.07
With crack MFLE	0.5	1	0.5	0 Material property Constant	

On the other hand, where the *L*_*c*_ grows, there are places with minimum properties where the displacements tend to concentrate, and there is also more fracture energy release at these places. Then the three exponents are linked at this point. Nonetheless, after the peak stress, the fractal exponent of strain goes to 1 because the crack is localized, while the fracture energy goes to 0.

**Figure 13:**
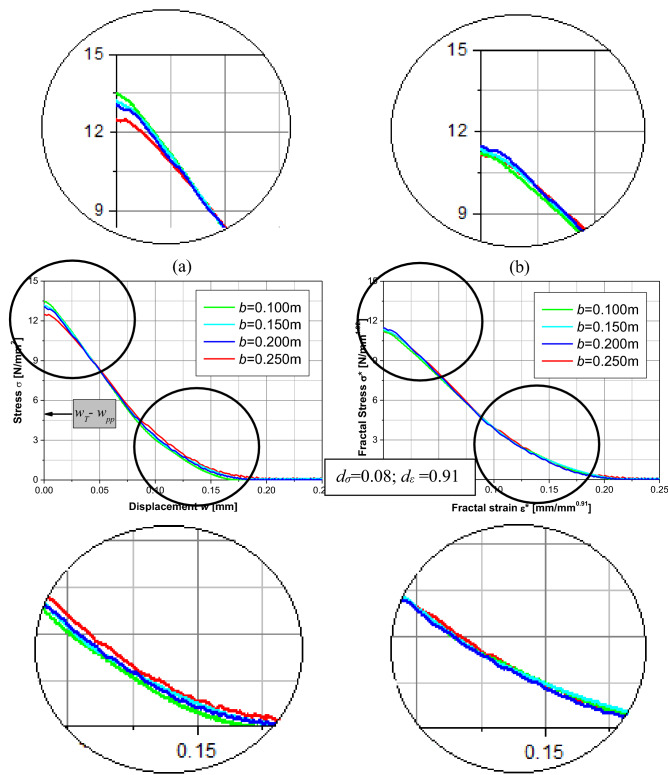
(**a**) Stress displacement curve, (**b**) fractal stress–strain curve for specimens simulated with *L*_*cx*_ = *L*_*cz*_ equal to 4 cm (8 times the length basic cubic module).

Figure 13a shows the stress versus *w*_*T*_*–w*_*pp*_ curve for specimens simulated with *L*_*cx*_ = *L*_*cz*_ equal to 4 cm (8 times the length of the basic cubic module). Figure 13b shows the fractal stress (${\sigma }^{*}=\sigma {b}^{{d}_{\sigma }}$) versus fractal strain (${\varepsilon }^{*}$) curve obtained for these specimens for *ε* ≥ *ε*_c_. In the fractal dominium, this relation is independent of the size.

It must be pointed out that the fractal stress–strain curve is obtained with the strain fractal exponent measured at failure. In the LDEM method, the unloading is elastic to the origin of the constitutive relationship (see Fig. 3b). For this reason, all the deformations only concentrate on one crack in the failure of one element of one LDEM module. As the size of the module is the same in all the specimens simulated, the final strain of all the specimens is related to the strain of one longitudinal element.

### Influence of the constitutive law

**Figure 14:**
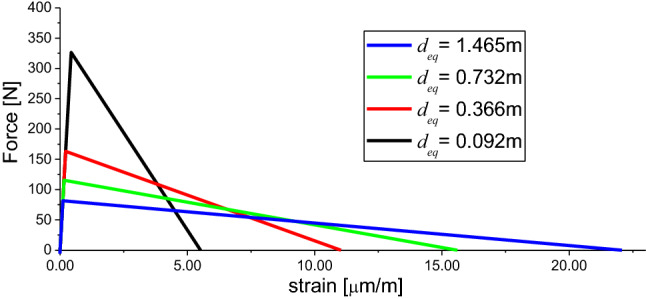
LDEM constitutive laws used in the analysis.

To analyze the influence of the constitutive law in the fractal exponent found in the simulation, the same constitutive law previously used and shown in Table 3 was used as a reference. In this study, only the *d*_*eq*_ is modified, that is the Young’s modulus *E*. The mean fracture energy related to size L, *μG*_*f*_; the coefficient of variation of *G*_*f*_ and its correlation length random field are the same as the previous one that was defined. Figure 14 shows the different mean constitutive laws utilized in this analysis.

**Figure 15:**
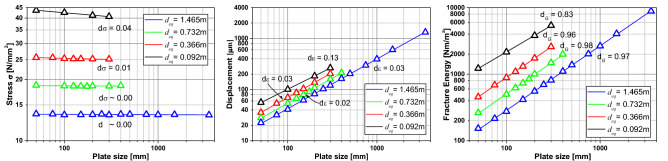
Ultimate global stress, ultimate and characteristic global displacement and fracture energy versus the specimen dimension for every constitutive law analyzed.

Figure 15 shows the mean of stress, displacement, and fracture energy versus the plate size for every constitutive law simulated. It is interesting to note here that the smaller the *d*_*eq*_, the bigger the fractal exponent of tensile, and strain will be and the smaller the fracture energy exponent (the sum of three exponents is still 1) will also be. These fractal exponents are calculated at the maximum stress, i.e., the peak of the stress–strain curve. Then, by changing the shape of constitutive law, it is possible to find values of fractal exponents more similar to the experimental ones.

The fractal stress–strain curve independent of the size is not built in this section because the response of the constitutive laws is brittle, then no stable branch is found in the simulation to show the invariability.

### Influence of the boundary condition

**Figure 16:**
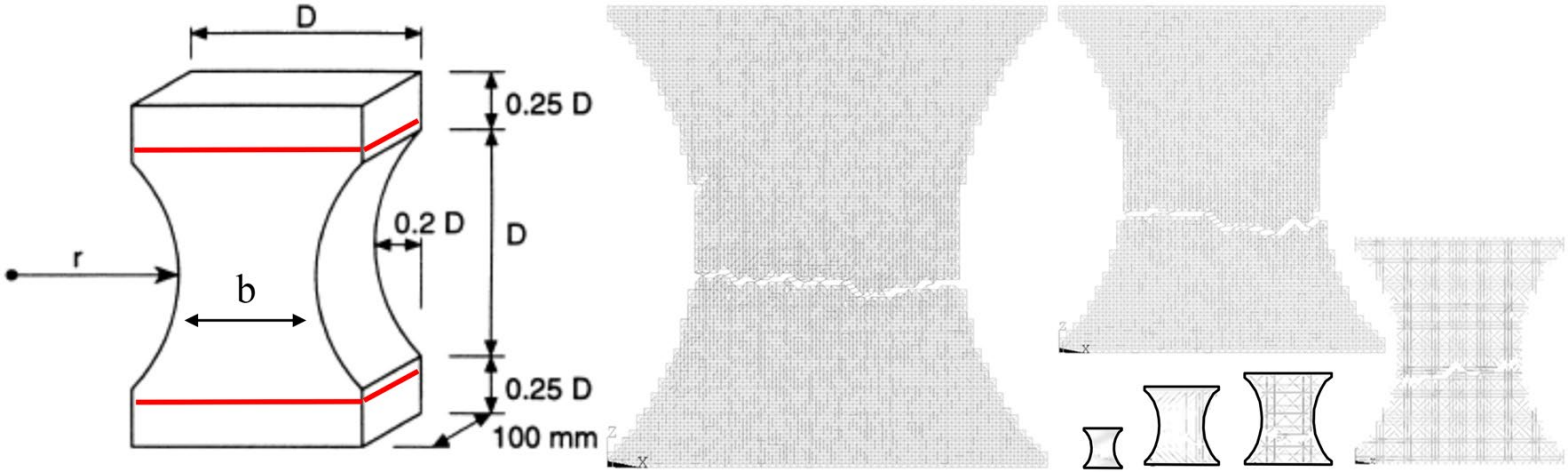
The relative size of the specimens and boundary conditions considered. The damage distribution and failure configuration of specimens of various sizes subjected to applied displacements inducing uniaxial tension. The characteristic specimen size b varies between 0.025 and 0.3 m.

**Figure 17:**
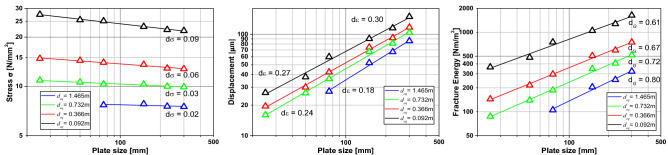
Ultimate global stress, ultimate and characteristic global displacement and fracture energy versus the specimen dimension for every constitutive law analyzed and with the geometry similar to^49^.

By changing the model’s geometry now, it is possible to show that the boundary condition alters the fractal exponent found. For this, plates on plane strain state with geometry similar to the one utilized by van Vliet and van Mier^49^ are used. The material properties are the same used in the previous examples. Figure 16 shows the geometry utilized, the specimens’ relative size, and the boundary conditions that were considered. The damage distribution and failure configuration of one of the six specimens simulated of various sizes subjected to applied displacements inducing uniaxial tension are also shown in this figure. The characteristic specimen size *b* varies between 0.025 and 0.3 m. The same constitutive laws presented in Fig. 17 are used in this study too.

Figure 17 shows the mean fractal exponent found in the simulations. When comparing Fig. 15 with Fig. 17, it is easy to note that the geometry of the plate changes the fractal exponent of stress, displacements and fracture energy. In this way, it is possible to conclude that the fractal parameters are not material properties, and that they change with the boundary conditions being applied to the test or the simulation.

## Conclusions

The numerical analysis of specimens subjected to tensile fracture employing the Lattice Discrete Element Method (LDEM) is initially described in the paper. Then a brief explanation of Carpinteri’s Fractal Law is also presented. The determination of the fractal dimension over a set of simulated LDEM rock specimens subjected to uniaxial tension is described next, and the calculus of the three exponents *d*_*σ*_, *d*_*ε*_, and *d*_*G*_ is finally presented. The influence of the statistical properties of the material, the constitutive law used, and the boundary conditions are also studied and analyzed in detail.

The results obtained show that the LDEM model captures the fractal law predictions. Therefore it remains a valid alternative to simulate fracture processes in quasi-brittle materials as well as for exploring different aspects of fracture in real solids. In addition, it is possible to point out the following conclusions:

- It is important to note, in agreement with the fractal law theory, that the sum of the three coefficients is approximately 1.
- The fractal exponent *d*_*G*_ obtained in the LDEM simulations with a correlation length equal to 1.5 mm was considerably smaller than the value expected in real materials. However, when this correlation length of fracture energy 3D random field is modified, the fractal exponent *d*_*G*_ obtained in the LDEM simulations can be altered, and it is more approximated to the experimental ones.
- The range interval obtained in LDEM of the *d*_*G*_ [0,1] was wider than the one identified in the Carpinteri fractal theory where *d*_*G*_ [0,0.5]. This point will be better analyzed in a future study. Nonetheless, an explanation of this difference is the fact that for the LDEM, the *G*_*f*_ input parameter is a scale-invariant parameter representing not only the specific energy spent in the localization region but also the energy in all types of dissipation that happen in the specimen in the damage process.
- The computation of *G*_f_^*^, *σ*_u_^*^ and *ε*_c_^*^ allows seeing that the values obtained with LDEM are consistent.
- The influence of the geometry specimen, boundary condition, constitutive Law parameters and the correlation length of the *G*_*f*_ random field produce sensible changes in the fractal coefficients. This fact indirectly shows the potential to adjust experimental results with LDEM using the fractal coefficients as a target.
- The possibilities of LDEM to represent the damage process help better understand the scale effect law using the theoretical tools as the fractal coefficients.

Therefore, LDEM is shown as an interesting alternative to study a simulation of a fracture in quasi-brittle materials and capture the size effect. In addition, its results are correlated with the fractal law theory.

## Data Availability

All data generated or analyzed during this study are included in this published article. Further detailed information on the datasets elaborated during the current study is available from the corresponding author and can be provided on reasonable request.
